# Evaluation of Primers OPF-01, P54, and 1253 to Identify *A. fumigatus*, *A. flavus*, and *A. niger* from Polymorphic Patterns Obtained by RAPD-PCR

**DOI:** 10.3390/pathogens13070574

**Published:** 2024-07-10

**Authors:** Carlos Alberto Castro-Fuentes, María Guadalupe Frías-De-León, María del Carmen González-Villaseñor, Esperanza Duarte-Escalante, Omar Esteban Valencia-Ledezma, Areli Martínez-Gamboa, Beatriz Meraz-Ríos, María del Rocío Reyes-Montes

**Affiliations:** 1 Posgrado en Ciencias Biológicas, Facultad de Medicina, Universidad Nacional Autónoma de México, Mexico City 04510, Mexico; castrofuenca@gmail.com; 2 Unidad de Investigación, Hospital Regional de Alta Especialidad de Ixtapaluca, IMSS-Bienestar. Calle Gustavo E. Campa 54, Col. Guadalupe Inn, Alcaldía Álvaro Obregón, Mexico City 01020, Mexico; esteban84valencia@gmail.com; 3 Unidad de Investigación Biomédica, Hospital Regional de Alta Especialidad de Ixtapaluca, IMSS-Bienestar. Calle Gustavo E. Campa 54, Col. Guadalupe Inn, Alcaldía Álvaro Obregón, Mexico City 01020, Mexico; magpefrias@gmail.com; 4 Instituto de Biología, Universidad Nacional Autónoma de México, Mexico City 04510, Mexico; mcgv@ib.unam.mx; 5 Departamento de Microbiología y Parasitología, Facultad de Medicina, Universidad Nacional Autónoma de México, Avenida Universidad 3000, Ciudad Universitaria, Coyoacán, Mexico City 04510, Mexico; dupe@unam.mx (E.D.-E.); bmerazr@hotmail.com (B.M.-R.); 6 Instituto Nacional de Ciencias Médicas y Nutrición Salvador Zubirán, Vasco de Quiroga 15, Belisario Domínguez Secc. 16, Tlalpan, Mexico City 14080, Mexico; areli_martinez@hotmail.com

**Keywords:** evaluation, *A. fumigatus*, *A. flavus*, *A. niger*, primers, RAPD-PCR

## Abstract

We evaluated the specificity of the primers OPF-01, P54, and 1253 to identify *A. fumigatus*, *A. flavus*, and *A. niger*, respectively, with the RAPD-PCR method. Eighty-two isolates belonging to the sections *Fumigati*, *Flavi*, and *Nigri* were used. The isolates were identified by phenotypic (macro- and micromorphology) and genotypic (partial sequences of the *BenA* gene) methods. The RAPD-PCR method was used to obtain polymorphic patterns with the primers OPF-01, P54, and 1253. The specificity of the polymorphic patterns of the isolates of each species was evaluated through the UPGMA clustering method and logistic regression model. All isolates of the genus *Aspergillus* were identified at the section level by macro- and micromorphology showing the typical morphology of the sections *Fumigati*, *Flavi*, and *Nigri*, and the species were identified by the construction of the phylogeny of the partial sequence of the *BenA* gene. The patterns’ polymorphic strains obtained with the primers OPF-01, P54, and 1253 for the isolates of *A. fumigatus*, *A. flavus*, and *A niger*, respectively, showed the same polymorphic pattern as the reference strains for each species. To verify the specificity of the primers, they were tested with other species from the sections *Fumigati*, *Flavi* and *Nigri.* The results support that the primers OPF-01, P54, and 1253 generate polymorphic patterns by RAPD-PCR species specific to *A. fumigatus*, *A. flavus*, and *A. niger*, respectively.

## 1. Introduction

Fungi of the genus *Aspergillus* are opportunistic pathogens that can cause aspergillosis in humans, and are acquired in the environment; they produce many small conidia, which are easily transported through the air and can be inhaled by a susceptible immunocompetent host or immunocompromised [1,2]. However, in immunocompetent hosts, they eliminate it efficiently, while, in immunocompromised hosts, the fungus can colonize the upper or lower respiratory tract and produce a wide range of clinical manifestations such as invasive pulmonary aspergillosis, chronic pulmonary aspergillosis, aspergillosis, allergic bronchopulmonary, and *Aspergillus* bronchitis, and induces various levels of disease severity [3].

The most clinically significant section is *Fumigati*, which comprises *A. fumigatus*, *A. lentulus*, and *A. udagawae*, among others [4]. The most relevant species outside the *Fumigati* section are *A. flavus*, *A. nidulans*, *A. terreus*, and *A. niger* [5]. In America and Europe, the species most frequently involved in human diseases is *A. fumigatus*, while *A. flavus* is gaining prevalence in some Asian countries [6]. Conventional procedures for the identification of *Aspergillus* spp. include pathology, direct examination, culture, and the detection of the galactomannan and (1→3)-β-D-glucan antigens, as well as polymerase chain reaction assays. In order to overcome the limitations of other methods [7,8], PCR has been included in the diagnosis of aspergillosis, with the aim of providing a more sensitive approach. However, it is important to note that PCR also has limitations [9].

In addition to these drawbacks, in recent years, invasive fungal diseases attributed to different species of *Aspergillus* have increased [10], which is why the need arises to identify the fungus at the species level, particularly because available antifungal agents differ in their spectrum of action [11,12]. Samson et al. [13] recommend a multiphase approach using a combination of phenotypic and sequencing methodologies for the identification of *Aspergillus* species; however, for many clinical laboratories, these types of assays are complicated and expensive. Therefore, new rapid and reliable identification strategies are necessary. In recent years, other molecular typing methods have been used to characterize fungal isolates and to delineate the relationship between strains; among these is random amplified polymorphic DNA (RAPD). This method has been used for the identification of fungi and it has been successfully applied to evaluate the genetic relationship of these, as is the case for *Sporotrhix* spp. [14], *Candida* spp. [15,16,17], and *Aspergillus* [18].

Likewise, [19] selected polymorphic patterns were obtained by RAPD-PCR through qualitative and quantitative analyses to differentiate the species *A. flavus*, *A. fumigatus*, *A. niger*, and *A. tubingensis*. The authors used 34 oligonucleotides to obtain polymorphic patterns and performed a qualitative analysis to select primers that exhibited species-specific patterns. For selection, a quantitative analysis was carried out using logistic regression, where a species-specific correlation of sensitivity and specificity was obtained for the primers: OPF-01 for *A. fumigatus*; P54 for *A. flavus*, and 1253 for *A. niger*. Thus, these quantitative methods to select species-specific primers showed their usefulness to identify some of the medically relevant species belonging to the *Aspergillus* genus. Therefore, the objective of this work was to evaluate the primers OPF-01, P54, and 1253 to identify *A. fumigatus*, *A. flavus*, and *A. niger* by RAPD-PCR.

## 2. Materials and Methods

### 2.1. Fungal Isolates

In this study, 82 *Aspergillus* isolates were used to test the efficiency of our method. These isolates were previously identified and characterized—specifically, 27 of *A. fumigatus*, 24 of *A. flavus*, 17 of *A. niger*, and 1 of *A. tubingensis*. The isolates were obtained from the collection of the Laboratorio de Micología Molecular del Departamento de Microbiología y Parasitología, Facultad de Medicina, Universidad Nacional Autónoma de México (UNAM). In addition, thirteen *Aspergillus* isolates were included that were characterized by macro- and micromorphology and sequencing of the *BenA* gene; these isolates were provided by the Instituto Nacional de Ciencias Médicas y Nutrición Salvador Zuribán (Table 1). In addition, five reference strains were used, obtained from the American Type Culture Collection (Manassas, VA, USA) (Table 2).

### 2.2. Identification of Sections of Aspergillus by Phenotypic and Genotypic Methods

#### Phenotypic Identification

The thirteen isolates isolates provided by the Instituto Nacional de Ciencias Médicas y Nutrición Salvador Zuribán were plated on potato-dextrose agar (PDA) medium (Bioxon, Mexico City, Mexico) at 28 °C for 4–7 days to identify their macro- and micromorphological characteristics, including color and colonial texture. The micromorphological characteristics of all isolates were analyzed using the microculture method of Riddell [20], following the procedures of Samson et al. [13].

### 2.3. Genotypic Identification

#### 2.3.1. DNA Extraction

From each culture of *Aspergillus* spp. seeded in PDA (Bioxon, CDMX, MX), a conidial suspension was obtained that was inoculated in tubes with 50 mL of YEPG culture medium (1% yeast extract, 2% peptone, and 2% dextrose) and incubated at 37 °C in an orbital shaker for three days until mycelial growth was observed. The mycelial biomass of each isolate was harvested by filtration and frozen at −20 °C until use. Fungal DNA was extracted using a DNeasy^®^ Plant Mini Kit (Qiagen, Austin, TX, USA). Total extracted DNA was quantified by 1% agarose gel electrophoresis and compared with different concentrations (10, 30, and 50 ng/µL) of phage λ (Gibco BRL^®^, San Francisco, CA, USA) stained with GelRed ™ nucleic acid gel stain 10,000× by Biotium (Fremont, CA, USA). Furthermore, it was also quantified by UV spectrophotometry using a NanoDrop 2000 spectrophotometer (Thermo Fisher Scientific, Waltham, MA, USA).

#### 2.3.2. Amplification of the Partial Sequence of the BenA Gene

PCR amplification of the *BenA* gene of the thirteen isolates was carried out as described by Glass and Donaldson [21], and the oligonucleotides used were Bt2a (5′-GGTAACCAAATCGGTGCTGCTTTC-3′) and Bt2b (5′-ACCCTCAGTGTAGTGACCCTTGGC-3′). A volume of 25 µL was used for the reaction mixture, with 10 mM of MgCl_2_, 100 µM of each dNTP, 1 U/µL Taq DNA polymerase, 10 µM of each primer, and 20 ng/µL of DNA. Amplification was carried out in a thermocycler Bio-Rad (Hercules, CA, USA) with the following conditions: 95 °C for 8 min; 35 cycles of 95 °C for 15 s; 55 °C for 20 s and 72 °C for 1 min; and a cycle of 72 °C for 5 min. The PCR products were sent for sequencing in both directions by Macrogen USA (Rockville, MD, USA), using the Sanger method.

#### 2.3.3. Sequence Analysis

For the analysis of the sequences, the BioEdit program ver. 7.2 was used (https://bioedit.software.informer.com/7.2/, accessed on 9 December 2023), which allowed us to manually corroborate the sequences obtained in the sequencing process (forward and reverse) of each sample and generate a consensus sequence. Each sequence was analyzed with the Basic Local Alignment Search Tool (BLAST) program ver. 1.4.9 [22] (https://blast.ncbi.nlm.nih.gov/Blast.cgi, accessed on 9 December 2023) to confirm its identity. Subsequently, the sequences were aligned with the MAFFT program ver. 7 (https://mafft.cbrc.jp/alignment/server/, accessed on 3 November 2023) [23], and the best evolutionary model applied to this alignment was chosen with the JModelTest 2 program ver. 1 (https://github.com/ddarriba/jmodeltest2, accessed on 8 January 2024) [24].

#### 2.3.4. Phylogenetic Analysis

For the construction of the phylogenetic tree, the sequences deposited in GenBank of the isolates already identified and the sequences of the isolates characterized in this study were included. The newly generated sequences were deposited in GenBank.

Phylogenetic analysis of the sequences was carried out using the maximum likelihood method. The support values of the internal branches were evaluated by a bootstrap method with 1000 repetitions (values equal to or greater than 70% were considered significant) and the GTR + G + I evolutionary model; the nearest neighbour interchange (NNI) heuristic method was applied. A maximum likelihood (ML) analysis was performed with the MEGA software v.10.1.7 [25]. Reference sequences obtained from GenBank were included in the phylogenetic analysis (Table 2).

### 2.4. Identification of A. fumigatus, A. flavus, and A. niger through Polymorphic Patterns Generated by RAPD-PCR with the Primers OPF-01, P54, and 1253, Respectively

#### Obtaining Polymorphic Patterns by RAPD-PCR

The RAPD-PCR method was used using the technique described by Kersulyte et al. [26] and Woods et al. [27]. Primers OPF-01 (5’-ACGGATTCTG-3’), P54 (5’-GGCGATTTTTGCCG-3’), and 1253 (5’-GTTTCCGCCC-3’) were tested as described below. Briefly, the RAPD-PCR reaction was carried out in a 25 μL volume containing 1X buffer, 2.5 mM MgCl2, 20 ng DNA, 200 μM of each dNTP (Applied Biosystems Inc., Waltham, MA, USA), 1 U Taq DNA polymerase (Invitrogen, Carlsbad, CA, USA), and 100 pmol/μL of each primer. The PCR conditions were as follows: 1 cycle of 7 min at 94 °C, followed by 45 cycles of 1 min at 92 °C, 1 min at 35 °C and 1 min at 72 °C, and a final extension of 5 min at 72 °C. The products were electrophoresed in 1.5% agarose gel stained with GelRed™ 10000X (Biotium). Images of the gels were captured on a Synoptics Photodocumenter (Syngene, San Diego, CA, USA).

### 2.5. Statistical Analysis

To evaluate the polymorphic patterns obtained with the primers OPF-01, P54, and 1253 specific to *A. fumigatus*, *A. flavus*, and *A. niger*, respectively, the Unweighted Pair Group Method with Arithmetic Mean (UPGMA) was used, and the logistic regression model through receiver operating characteristics (ROC) curves.

#### 2.5.1. UPGMA

RAPD markers were visually recorded, manually coded, and translated into binary data that indicated either their presence (1) or absence (0). The genetic similarity between isolates was calculated with the Jaccard index. Genetic relationships among isolates were assessed using the mean of the Unweighted Pair Group Method with Arithmetic Mean (UPGMA) and were carried out using the NTSYS-PC program (version 2.0, Exeter Software, New York, NY, USA) [28].

#### 2.5.2. Logistic Regression Model

From the polymorphic patterns obtained with the primers OPF-01, P54, and 1253 specific to *A. fumigatus*, *A. flavus*, and *A. niger*, respectively, a database was built considering the number of bands per isolate, the molecular size, and the intensity of each one, according to the following ranges: 0.5 (very faint); 1.0 (dim); 2.0 (intense); and 3.0 (very intense). The database obtained was used to build logistic regression models considering as dependent variables the band number, molecular size, and intensity, while the species of the fungus was considered as an independent variable. These variables were analyzed in the JMP^®^Pro 13 program (SAS Institute Inc., Cary, NC, USA). Subsequently, the significance of the logistic regression models and the study variables were evaluated to select the model that presented the highest value of sensitivity vs. specificity using the ROC curves. The values of the ROC curves obtained in this study were compared with the values of the ROC curves obtained by Valencia-Ledezma et al. [19]. Primers were considered specific with an area under the curve (AUC) value greater than 0.9 in sensitivity vs. specificity. The model obtained from the selected primers generated a mathematical equation that allowed the estimation of the most probable species.

## 3. Results

### 3.1. Identification of Aspergillus Species by Phenotypic and Genotypic Methods

The thirteen isolates of *Aspergillus* presented the typical macro- and micromorphology described for the respective species. Species from the following sections were identified: section *Fumigati* (32-16078, 459, 73-1904, and 3IC isolates), section *Flavi *(172 and 181 isolates), and section *Nigri* (219, 232, 227, 213, 203, 205, and 221 isolates).

To confirm the *Aspergillus* species, a phylogenetic analysis was carried out with the sequences of the *BenA* gene fragment of all the isolates studied. All isolates were grouped with the reference strains corresponding to the different *Aspergillus* species with a bootstrap of 96-99%. The tree formed 11 groups: Group I includes the isolates of the section *Nigri* and is divided into three subgroups: in subgroup Ia are the isolates 219 and the strain WB326 ATCC that are grouped with the reference strains of *A. niger* (MT410073.1, MT410068.1, MT410066.1, MT410071.1, MT410076.1, MT410075.1, MT410078.1, MT410079.1, OM892858.1, MT410061.1, OM892861.1, OM892859.1, MT410063.1, MT410067.1, MT410062.1, MT410069.1, and OM892860.1); in subgroup Ib are the isolates 205, 221, and 203 that are grouped with the reference sequences of *A. luchuensis* (MH063939.1 and PP315916.1); and in subgroup Ic are the isolates 227, 213, and 232, and strain 1004 ATCC that were grouped with the reference strains of *A. tubingensis* (MT410082.1, MT410084.1, OM892870.1, OM892872.1, OM892873.1, OM892871.1, MT410083.1, and EF661086.1). In group II, there are the reference sequences of the section *Candidi* (HE661604.1 and MN969367.1). Group III corresponded to the isolates of the section *Flavi*, which is divided into four subgroups: in subgroup IIIa, there are isolates 172 and 181, which are grouped with the reference strain of *A. tamari* (EF661474.1); in subgroup IIIb are the reference strains *A. arachidicola* (EF203158.1), *A. parasiticus* (EF66148.1), and *A. austwickii* (MG517702.1); in subgroup IIIc is the strain 96430-2 ATCC which is grouped with the reference sequences of *A. flavus* (OQ560611.1, OQ560607.1, OQ560593.1, OM892869.1, OQ560592.1, OQ560598.1, OQ560603.1, OQ560600.1, OM892868.1, MT347711.1, OQ560586.1, OQ560609.1, OQ560584.1, OQ560583.1, OQ560587.1, OQ560597.1, OQ560588.1, OQ560605.1, OQ560606.1, OQ560595.1, MT347712.1, EF661485.1, MT347713.1, and OQ560608.1). In group IV, there are the reference sequences of the section *Terrei* (EF669524.1 and MT472459.1). In group V are the reference sequences of the section *Flavipides* (EU014085.1 and EU014086.1). In group VI, there are the reference sequences of the section *Usti* (FJ531179.1 and EF652331.1). In group VII, there are the reference sequences of the section *Nidulantes* (EF652248.1, EF652274.1, and EF652266.1). In group VIII, there are the reference sequences of the section *Clavati* (EF669789.1 and MK451093.1). Group IX contain the isolates of the section *Fumigati*, and they are divided into four subgroups: in subgroup IXa are reference sequences of *A. udagawae* (LT796063.1 and MK451259.1); the subgroup IXb contains isolates 31C and 73-1904, which are grouped with the *A. hiratsukae* reference sequence (AF057324.1); the subgroup IXc contains isolates 459 and MYA3566 ATTC, which are grouped with the *A. lentulus* reference sequences LR584265.1 and EF669825.1; in subgroup IXd are the isolates 32-16076 and MYA3626 ATTC that grouped with the reference sequences of *A. fumigatus* (EF669791.1, MN637737.1, OM892862.1, OM892863, MN637727.1, MN637747.1, MT347702.1, OM892865.1, MN637734.1, MN637704.1, MN637724.1, OM892864.1, MN637732.1, MN637767.1, MN637741.1, MN637733.1, MT347701.1, MT347703.1, MN637749.1, MT196114.1, MN637755.1, MT196113.1, MN637757.1, MN637765.1, MN637777.1, MN637773.1, MN637736.1, and MN637779.1). In group X, there are the reference sequences of the *Aspergillus* section (EF651911.1 and EF651891.1). In group XI, there are the reference sequences of the section *Circumdati* (LS423510.1 and EF661329.1) (Figure S1).

### 3.2. RAPD-PCR with Primers OPF-01, P54, and 1253 to Generate Polymorphic Patterns Specific to A. fumigatus, A. flavus, and A. niger, Respectively

**Figure 1:**
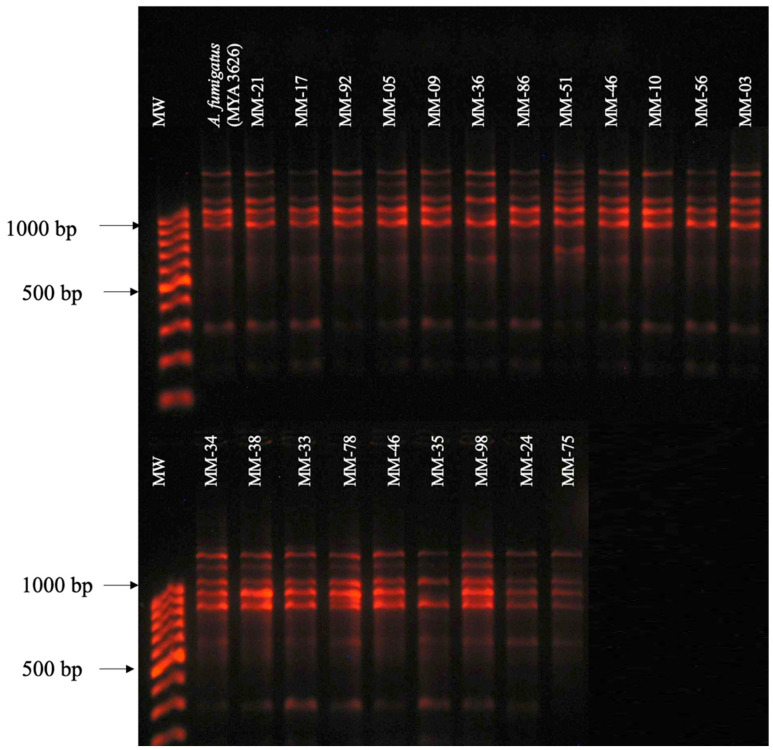
Polymorphic patterns of *A. fumigatus* isolates were obtained by RAPD-PCR with primer OPF-01. MW: Molecular weight marker 100 bp DNA ladder (Invitrogen by Life Technologies). Conditions were as described in the Materials and Methods section.

Of all the isolates identified as *A. fumigatus*, 22 of them showed a polymorphic pattern of nine bands of 180, 300, 650, 900, 950, 1000, 1100, 1300, and 1500 bp obtained with the primer OPF-01, which coincided with the polymorphic pattern of the reference isolate of *A. fumigatus* (MYA3626/ATCC) (Figure 1).

**Figure 2:**
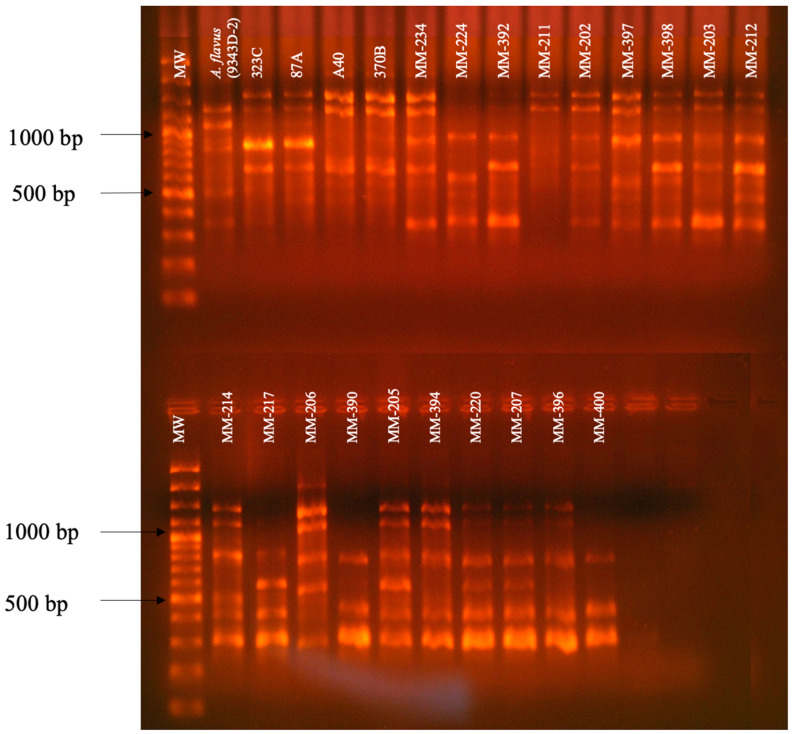
Polymorphic patterns of *A. flavus* isolates were obtained by RAPD-PCR with primer P54. MW: Molecular weight marker 100 bp DNA ladder (Invitrogen by Life Technologies). Conditions were as described in the Materials and Methods section.

The polymorphic pattern obtained with the primer P54 was similar for *A. flavus* isolates, formed by 12 bands located between 200 bp–1500 bp that coincided with the polymorphic pattern of the reference isolate of *A. flavus* (9343D-2/ ATCC) (Figure 2).

**Figure 3:**
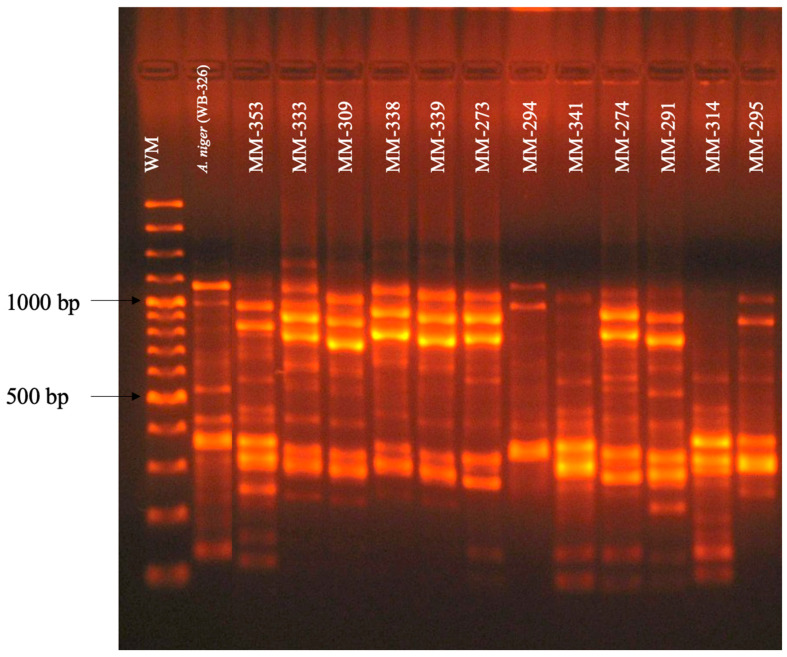
Polymorphic patterns of *A. niger* isolates were obtained by RAPD-PCR with primer 1253. MW: Molecular weight marker 100 bp DNA ladder (Invitrogen by Life Technologies). Conditions were as described in the Materials and Methods section.

The polymorphic pattern obtained with the primer 1253 was similar for *A. niger* isolates, and was characterized by the presence of 17 bands in a range of 100, 150, 180, 200, 220, 250, 300, 380, 400, 480, 520, 600, 780, 820, 900, 1000, and 1080 bp that coincided with the polymorphic pattern of the reference isolate of *A. niger* (WB-326-ATCC) (Figure 3).

### 3.3. Evaluation of the Specificity of Primers OPF-01, P54, and 1253

To evaluate the specificity of the primers OPF-01, P54, and 1253, they were tested by RAPD-PCR with other *Aspergillus* species from the sections *Fumigati*, *Flavi*, and *Nigri*, respectively. Furthermore, to corroborate the similarity of the intra-species polymorphic patterns, a dendrogram was constructed through the UPGMA method, for the isolates from each section (*Fumigati*, *Flavi*, and *Nigri*).

**Figure 4:**
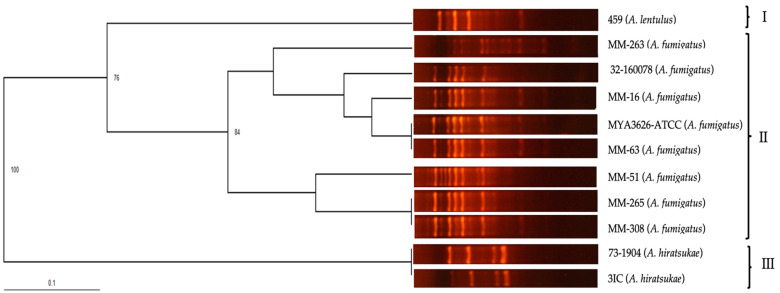
UPGMA dendrogram calculated from the comparison of polymorphic patterns obtained by RAPD-PCR of *Aspergillus* section *Fumigati* isolates, obtained with primer OPF-01.

The polymorphic pattern obtained with species from the section *Fumigati* was the same for *A. fumigatus* isolates and different for other species from the section *Fumigati*. The dendrogram obtained for isolates from the section *Fumigati* corroborated the specificity of the first OPF-01 through the following grouping: the tree showed three groups: group I included the *A. lentulus* isolate, group II included the *A. fumigatus* isolates, and group III included the *A. hiratsukae* isolates (Figure 4).

**Figure 5:**
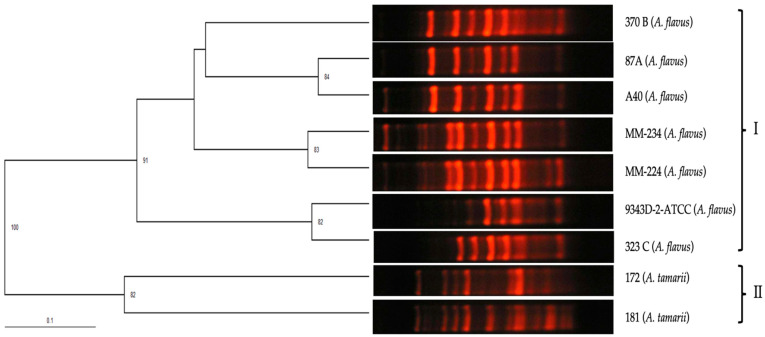
UPGMA dendrogram calculated from the comparison of polymorphic patterns obtained by RAPD-PCR of *Aspergillus* section *Flavi* isolates, obtained with primer P54.

The polymorphic pattern obtained with species from the section *Flavi* was the same for *A. flavus* isolates and different for the *A. tamari* species, included in the section *Flavi*. The dendrogram obtained for isolates from the section *Flavi* corroborated the specificity of the primer P54 through the following grouping: the tree showed two groups: group I included the *A. flavi* isolates and group II the *A. tamari* isolates (Figure 5).

**Figure 6:**
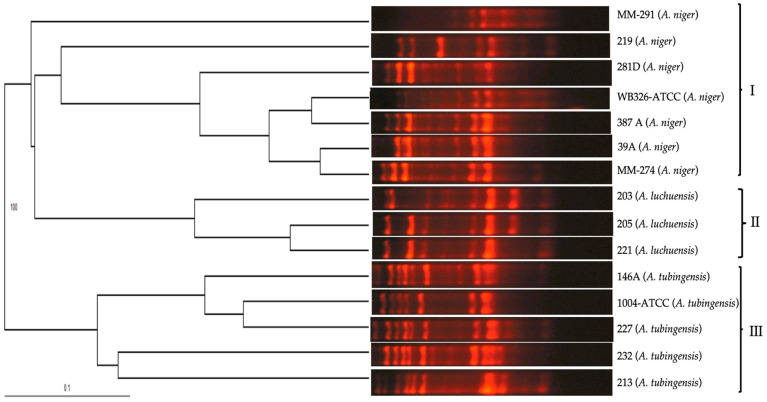
UPGMA dendrogram calculated from the comparison of polymorphic patterns obtained by RAPD-PCR of *Aspergillus* section *Nigri* isolates, obtained with primer 1253.

The polymorphic pattern obtained with species from the section *Nigri* was the same for *A. niger* isolates and different for other species, included in the section *Nigri*. The dendrogram obtained for isolates from the section *Nigri* corroborated the specificity of the primer 1253 through the following grouping: the tree showed three groups: group I included the isolates of *A. niger*, group II included isolates of *A. luchuensis*, and the group III the *A. tubingensis* isolates (Figure 6).

### 3.4. Sensitivity and Specificity of Primers

**Figure 7:**
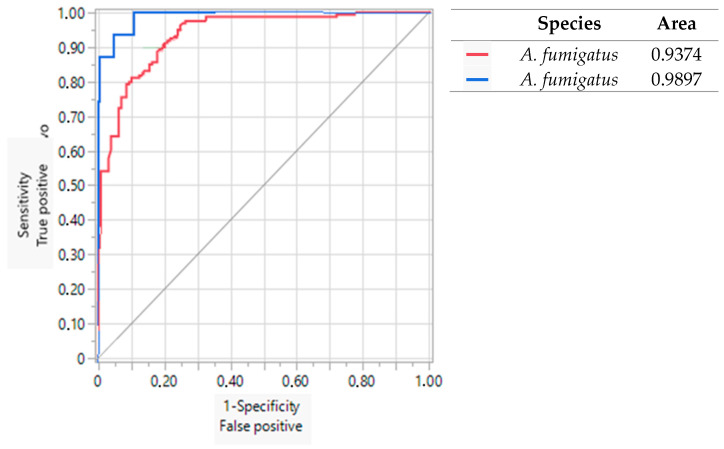
Evaluation of the specificity vs. sensitivity of the polymorphic patterns of *A. fumigatus* obtained with the primer OPF-01 through the ROC curve. (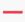): Area under the curve of *A. fumigatus* isolates tested in this study. (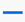): Area under the curve obtained by Valencia-Ledezma et al. [19].

**Figure 8:**
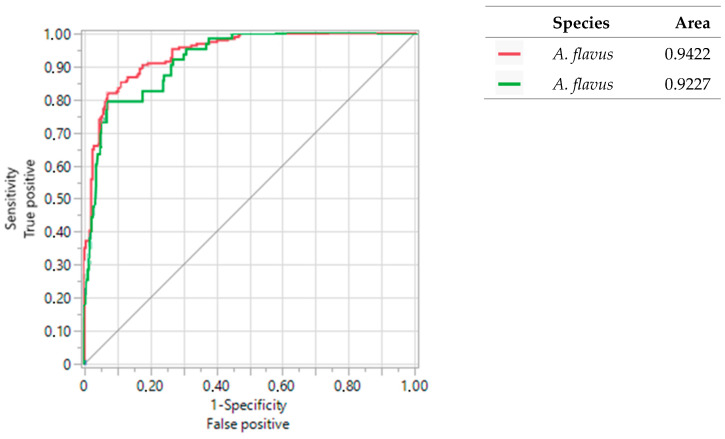
Evaluation of the specificity vs. sensitivity of the polymorphic patterns of *A. flavus* obtained with the primer P54 through the ROC curve. (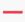): Area under the curve of *A. flavus* isolates tested in this study (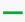): Area under the curve obtained by Valencia-Ledezma et al. [19].

**Figure 9:**
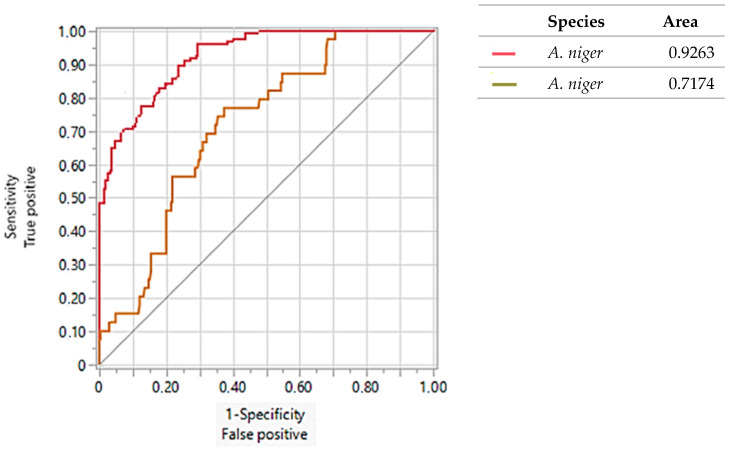
Evaluation of the specificity vs. sensitivity of the polymorphic patterns of *A. niger* obtained with the primer 1253 through the ROC curve. (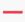): Area under the curve of *A. niger* isolates tested in this study (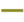): Area under the curve obtained by Valencia-Ledezma et al. [19].

The values of the ROC curves obtained in this study were compared with the values of the ROC curves obtained by Valencia-Ledezma et al. [19]. Figure 7, Figure 8 and Figure 9 show the results of the ROC curves obtained for OPF-01, P54, and 1253 with isolates of *A. fumigatus*, *A. flavus*, *A. niger*, and reference strains. With the primer OPF-01 for *A. fumigatus*, a correlation was observed between the area under the curve values of 0.93 for the isolates of this study with the values of 0.98 for those obtained by Valencia-Ledezma et al. [19] (Figure 7). With the primer P54 for *A. flavus*, a correlation was observed between the area under the curve values of 0.94 for the isolates of this study with the values of 0.92 for those obtained by Valencia-Ledezma et al. [19] (Figure 8). The primer P54 demonstrated specificity for *A. flavus* with an area under the curve value of 0.94 for the tested isolates and a species-specific correlation with an area under the curve value of 0.92, while the primer 1253 demonstrated specificity for *A. niger* with an area under the curve value of 0.92 for the tested isolates and a species-specific correlation with an area under the curve value of 0.72 for those obtained by Valencia-Ledezma et al. [19] (Figure 9).

## 4. Discussion

The importance of identifying the species of the genus *Aspergillus* lies in the fact that, in recent years, numerous cryptic species have been described within the sections *Fumigati*, *Nigri*, *Flavi*, and *Terrei*, mainly, which can cause aspergillosis, both in humans as in animals, and some of these species have a different susceptibility to the antifungals available for treatment. Although the incidence of the new species is not as high as that of *A. fumigatus*, its correct identification is essential in order to implement specific therapeutic strategies for each patient [29]. Therefore, this work describes the evaluation of the primers OPF-01, P54, and 1253, to generate species-specific polymorphic patterns by RAPD-PCR that allow the discrimination of the species *A. fumigatus*, *A. flavus*, and *A. niger*, respectively. This method represents an alternative approach to species identification.

To carry out the evaluation of the primers, a phylogeny was initially constructed with sequences of the *BenA* gene of each isolate, to corroborate the identity of the *Aspergillus* species included in the present study (Figure S1). The selection of the *BenA* gene was because it has been shown to be useful for phylogenetic relationship studies of *Aspergillus* and related species [30,31], since it is a conserved, slowly evolving gene with a high degree of variability between species. In addition, the *BenA* gene has also been used to distinguish cryptic *Aspergillus* species [32].

Subsequently, the DNA of the isolates of *A. fumigatus*, *A. flavus*, and *A. niger* was analyzed through the RAPD-PCR technique with the primers OPF-01, P54, and 1253. The profiles obtained by the RAPD-PCR with these primers were very informative and generated polymorphic patterns that coincided with those obtained with the reference strains of *A. fumigatus*, *A. flavus*, and *A. niger*, respectively, allowing their identification.

Likewise, to corroborate the specificity of these primers, other isolates of medical importance belonging to the sections *Fumigati*, *Flavi*, and *Nigri* were used, through RAPD-PCR. The results confirmed that, with the primers OPF-01, P54, and 1253, species-specific polymorphic patterns were obtained for *A. fumigatus*, *A. flavus* and *A. niger*, while different polymorphic patterns were obtained for other species from the sections *Fumigati, Flavi*, and *Nigri*. Likewise, to demonstrate the specificity of these primers, the polymorphic patterns were analyzed using the UPGMA method. The three dendrograms (Figure 7, Figure 8 and Figure 9) grouped the species of *A. fumigatus*, *A. flavus*, and *A. niger* into groups separate from other species, confirming the specificity of the three primers.

Furthermore, the results of the logistic regression method through the values of the ROC curves supported the specificity of the OPF-01, P54, and 1253 primers, since they presented similar area under the curve values between the isolates tested in this study and those obtained by [19]. Therefore, these primers are useful in the identification of the species of *A. fumigatus*, *A. flavus*, and *A. niger*.

With the above, it is verified that the RAPD-PCR technique can be used as a tool that allows the identification of *Aspergillus* species. The use of species-specific primers for species identification has been used in other fungi with good results, such as the use of the universal primer T3B in PCR fingerprinting, to differentiate between strains of *C. albicans* and *C. dubliniensis* [16,17], and it was also used to distinguish isolates of *C. albicans*, *C. glabrata*, *C. parapsilosis*, *C. tropicalis*, *C. guilliermondii*, *C. krusei*, and *C. lusitaniae*, which are the species most common in clinical cases [15]. Likewise, the primer T3B has also been used in PCR fingerprinting to distinguish the species of the *Sporothrix* complex (*S. brasiliensis*, *S. globosa*, *S. Mexicana*, and *S. schenckii*) [14]. It was also used by Pena et al. [33], who applied PCR restriction fragment length polymorphisms (PCR-RFLPs) and random amplification of polymorphic DNA molecular markers (RAPD) to characterize a set of clinical strains of *A. fumigatus* from Argentina and Brazil. The results showed that the strains from Argentina and Brazil grouped with the sensu stricto reference strains of *A. fumigatus*, forming a single group, regardless of their source of isolation and geographical origin. Therefore, it is considered that these primers are specific to *A. fumigatus*. While Hong et al. [34] also used the RAPD-PCR technique with the primers PELF and URP1, these primers showed specific patterns and discriminated between closely related species, between clinical and environmental isolates of *Aspergillus*. Likewise, the RAPD-PCR technique has demonstrated a discriminatory capacity to identify different species of *Aspergillus* as reported by Kermani et al. [18], who used seven primers, which conferred specific patterns for the species. This background supports the usefulness of RAPD-PCR with species-specific primers to be used as an identification tool for the species of *A. fumigatus*, *A. flavus*, and *A. niger*; since it is a simple molecular tool, reliable, fast, and economical, it also requires less technical experience than sequencing. It has the advantage of identifying a great variety of species using the same methodology. Furthermore, these advantages are precious in a laboratory with limited facilities, making it an ideal identification methodology for clinical mycology laboratories; however, the main disadvantage of this method is the reproducibility of RAPDs. However, it has been shown that the method can be reproducible under carefully controlled conditions, which is why the strict standardization of PCR conditions is required. It should also be taken into account, as a general rule, only to consider the bands of polymorphic DNA that are observed in repeated amplifications and that involve different DNA preparations, and whether their presence or absence is not affected when the amount of the DNA target is doubled. Other factors that could cause a variation in the polymorphic DNA banding patterns are the change in the thermocycler machine and the source of the Taq DNA polymerase, so it is recommended that we use the same thermocycler, as well as the same batch of reactive [35].

## 5. Conclusions

The cryptic species reported within the genus *Aspergillus* present a different susceptibility to antifungals. Therefore, it is of the utmost importance to identify the species that cause infection, through a simple and easy-to-implement method at a relatively low cost for the diagnosis of aspergillosis, such as RAPD-PCR.

The primers OPF-01, P54, and 1253 generate polymorphic patterns by RAPD-PCR species specific to *A. fumigatus*, *A. flavus*, and *A. niger*, respectively.

However, this method’s limitation is that it cannot be applied directly to clinical samples since it is necessary to isolate the fungus. However, it serves to resolve cases in which the observation of the typical structures (aspergillary heads) is not achieved.

## Figures and Tables

**Table 1:** *Aspergillus* isolates belonging to the sections *Fumigati*, *Flavi*, and *Nigri*.

Specie	Isolate/GenBank Accession No.	Geographical Origin
*A. fumigatus*	MM-34/MN637747	Mexico
*A. fumigatus*	MM-92/MN637737	Mexico
*A. fumigatus*	MM-09/MN637704	Mexico
*A. fumigatus*	MM-36/MN637733	Mexico
*A. fumigatus*	MM-46/MN637727	Mexico
*A. fumigatus*	MM-10 /MN637732	Mexico
*A. fumigatus*	MM-38/MN637734	Mexico
*A. fumigatus*	MM-33/MN637724	Mexico
*A. fumigatus*	MM-46 /MN637727	Mexico
*A. fumigatus*	MM-35/MN637736	Mexico
*A. fumigatus*	MM-98/MN637741	Mexico
*A. fumigatus*	MM-17 /MT196113	Argentina
*A. fumigatus*	MM-05/MN637755	Argentina
*A. fumigatus*	MM-86 /MN637757	Argentina
*A. fumigatus*	MM-03 /MN637749	Argentina
*A. fumigatus*	MM-78 /MN637765	Argentina
*A. fumigatus*	MM-24 /MN637767	Argentina
*A. fumigatus*	MM-75/MN637773	Argentina
*A. fumigatus*	MM-21/ OM892865	Argentina
*A. fumigatus*	MM-16/ OM89286	Argentina
*A. fumigatus*	MM-51//MN637777	Francia
*A. fumigatus*	MM-56 /MN637779	Francia
*A. fumigatus*	MM63/MT196114	Peru
*A. fumigatus*	MM-263/MT347701.1	Cuba
*A. fumigatus*	MM-265/MT347702.1	Cuba
*A. fumigatus*	MM-308/MT347703.1	Cuba
*A. fumigatus*	21 INCMNSZ (32-16076)	Mexico
*A. lentulus*	18 INCMNSZ (459)	Mexico
*A. hiratsukae*	19 INCMNSZ (73-1904)	Mexico
*A. hiratsukae*	17 INCMNSZ (3IC)	Mexico
*A. flavus*	323C/OM89286	ND
*A. flavus*	87A/OM892858	ND
*A. flavus*	A40/OM892868	ND
*A. flavus*	370B/OM892869	ND
*A. flavus*	MM-234/MT347712.1	ND
*A. flavus*	MM-224/MT347711.1	ND
*A. flavus*	MM-392/OQ560605	Mexico
*A. flavus*	MM-211/OQ560592	Mexico
*A. flavus*	MM-202/OQ560583	Mexico
*A. flavus*	MM-397/OQ560608	Mexico
*A. flavus*	MM-398/OQ560609	Mexico
*A. flavus*	MM-203/OQ560584	Mexico
*A. flavus*	MM-212/OQ560593	Mexico
*A. flavus*	MM-214/OQ560595	Mexico
*A. flavus*	MM-217/OQ560597	Mexico
*A. flavus*	MM-206/OQ560587	Mexico
*A. flavus*	MM-390/OQ560603	Mexico
*A. flavus*	MM-205/OQ560586	Mexico
*A. flavus*	MM-394/OQ560606	Mexico
*A. flavus*	MM-220/OQ560600	Mexico
*A. flavus*	MM-207/OQ560588	Mexico
*A. flavus*	MM-396/OQ560607	Mexico
*A. flavus*	MM-400/OQ560611	Mexico
*A. flavus*	MM-218/OQ560598	Mexico
*A. tamarii*	2 INCMNSZ (172)	Mexico
*A. tamarii*	3 INCMNSZ (181)	Mexico
*A. niger*	MT410061	ND
*A. niger*	387A/OM892858	Mexico
*A. niger*	39A/OM892859	Mexico
*A. niger*	MM-353/MT410079	Cuba
*A. niger*	MM-333/MT410073	Cuba
*A. niger*	MM-309/MT410068	Cuba
*A. niger*	MM-338/MT410075	Cuba
*A. niger*	MM-339/MT410076	Cuba
*A. niger*	MM-273/MT410061	Cuba
*A. niger*	MM-294/MT410066	Cuba
*A. niger*	MM-274/MT410062	Cuba
*A. niger*	MM-291/MT410063	Cuba
*A. niger*	MM-341/	ND
*A. niger*	MM-314/	ND
*A. niger*	6 INCMNSZ (219)	Mexico
*A. niger*	MM-295/MT410067	Cuba
*A. niger*	281D/OM892871	Mexico
*A. tubingensis*	146A/OM892872	Mexico
*A. tubingensis*	10 INCMNSZ (232)	Mexico
*A. tubingensis*	13 INCMNSZ (227)	Mexico
*A. tubingensis*	7 INCMNSZ (213)	Mexico
*A. luchuensis*	1 INCMNSZ (203)	Mexico
*A. luchuensis*	8 INCMNSZ (205)	Mexico
*A. luchuensis*	9 INCMNSZ (221)	Mexico

ND: Undetermined.

**Table 2:** ATCC reference strains.

Species	Strain ATCC
*A. fumigatus*	MYA3626
*A. flavus*	9343D-2
*A. lentulus*	3566
*A. niger*	WB326
*A. tubingensis*	1004

## Data Availability

The data are contained within the article and Supplementary Materials.

## Supplementary Materials

The following supporting information can be downloaded at: https://www.mdpi.com/article/10.3390/pathogens13070574/s1, Figure S1: Phylogenetic tree based on the *BenA* gene sequence.
