# Supplementary material for: Evaluation of Primers OPF-01, P54, and 1253 to Identify A. fumigatus, A. flavus, and A. niger from Polymorphic Patterns Obtained by RAPD-PCR
Source: Pathogens. 2024 Jul 10;13(7):574. doi: 10.3390/pathogens13070574 (PMC11280055; doi:10.3390/pathogens13070574)
Supplement: Supplementary file 1 [file pathogens-13-00574-s001.zip › pathogens-3070052-SI.pdf]

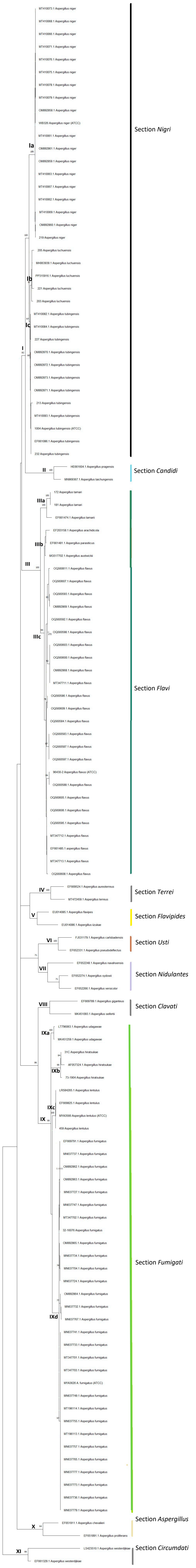

Figura S1. Phylogenetic tree based on the *BenA* gene sequence. The maximum likelihood analysis (ML) was performed with MEGA software v.10.1.7, using the substitution model general time reversible (GTR) model and gamma distributed (+G) with invariant sites (+I) (= GTR + G + I). All positions containing gaps and missing data were included for analysis. Clade supports were calculated based on 1000 bootstrap re-samplings.
